# Are There Non-Responders to the Ergogenic Effects of Caffeine Ingestion on Exercise Performance?

**DOI:** 10.3390/nu10111736

**Published:** 2018-11-12

**Authors:** Jozo Grgic

**Affiliations:** Institute for Health and Sport (IHES), Victoria University, Melbourne 3001, Australia; jozo.grgic@live.vu.edu.au

**Keywords:** individual responses, ergogenic aid, supplement, did not respond, responders

I have read with interest the recent review paper by Southward and colleagues [1]. While acknowledging that this was not the main focus of the paper, the authors attempted to estimate the average number of non-responders to caffeine ingestion in studies that investigated the effects of caffeine on time-trial performance [1]. Southward and colleagues [1] suggested that there might up to 33% of those who do not enhance performance following caffeine ingestion (i.e., non-responders). The authors came to this estimate by examining the change in performance following caffeine and placebo ingestion from the individual responses in several studies that reported these data. They used an approach where each participant that did not perform better on caffeine (as compared to placebo) was deemed as a non-responder. However, the authors did not consider that some of these individual differences between the caffeine and placebo conditions might have been merely an error of the measurement of the performance tests and not a true lack of response. Therefore, I believe that some additional discussion is needed to avoid confusion on this topic and to clarify the interpretation of these results.

## 1. Reliability of the Exercise Protocol

Reliability refers to the reproducibility of values of a given test [2]. In sport and exercise science, reliability is commonly determined by the error of measurement using the coefficient of variation (expressed as the percentage of the mean) [2]. If we examine the same set of studies as Southward et al. [1], while factoring in the coefficient of variation for the performance tests (for studies that provided these data), it becomes clear that the percentage of those that did not enhance performance following caffeine ingestion reduces from the initially suggested 33% to only 5% (Table 1) [3,4,5,6,7,8,9,10,11,12,13,14,15,16,17,18,19,20,21]. While such an amount is low, I would further question if there are really non-responders to the ergogenic effects of caffeine ingestion on exercise performance or if such inferences are an over-extrapolation of the results from the current body of evidence.

## 2. Using Multiple Exercise Tests When Examining the Effects of Caffeine

We have reported that caffeine ingestion in the dose of 6 mg/kg enhanced lower-body one-repetition maximum (1RM) strength and upper-body ballistic performance [22]. A scrutiny of the individual data from our study shows the problems when classifying responders and non-responders solely based on the results from one test. The figures provided in that paper indicate that participant number 8 experienced a 7% decrease in 1RM strength following caffeine ingestion (as compared to placebo). In contrast to the results for strength, the same participant experienced a 4% increase in upper-body ballistic performance following the ingestion of caffeine. If we were to present findings from only one test of performance the same participant can be classified as a non-responder to caffeine (based on the strength data) or as a responder (based on the ballistic performance data). Therefore, it becomes clear that caffeine might not enhance performance in one test while being effective in another. Classifying an individual as a non-responder to caffeine while focusing on the results from only one performance test may undermine the effects of caffeine that the same individual might experience in a different exercise task. These concepts can be juxtaposed with the findings by Churchward-Venne et al. [23] who reported no non-responders to a resistance training program when using several different test of performance (e.g., strength assessment in different exercise, chair-raise time, changes in lean body mass) given that each participant improved in at least some of the employed tests.

## 3. Using the Same Exercise Test with Different Doses of Caffeine

Jenkins et al. [24] tested the effects of 1, 2, and 3 mg/kg of caffeine on cycling performance. On average, their results indicated that only a 2 mg/kg dose of caffeine was effective for acute increases in cycling performance. However, the individual data presented in this study provided some very insightful findings. For instance, participant number 7 had a 6% decrease in performance following the ingestion of 1 mg/kg of caffeine. If Jenkins et al. [24] only used this dose of caffeine, this individual would be classified as a non-responder. However, in the 3 mg/kg caffeine condition, this participant improved cycling performance by +10%, and, if the researchers used only this dose of caffeine, the same participant would be considered as a high-responder to caffeine. While there are participants from the study by Jenkins et al. [24] that did not improve performance with any of the three caffeine doses, it is possible that higher doses of caffeine (e.g., 4–6 mg/kg) would elicit an acute improvement in performance even in these individuals. Some of these initial observations suggest that if an individual does not respond to a specific dose of caffeine, it would be erroneous to classify him as a non-responder given that a different dose (higher or lower) might be highly effective even in the same exercise task.

## 4. Repeated Testing Using the Same Exercise Test and the Same Dose of Caffeine

Astorino et al. [25] tested the same group of participants, using the same exercise test (cycling time-trial), and the same caffeine dose (5 mg/kg) on two different occasions. The results obtained by Astorino et al. [25] suggested that the effects of caffeine are repeatable in the majority of the participants as most tended to improve performance on both caffeine conditions. More importantly, the individual data presented in that study suggests that one participant improved cycling performance only in the second administration of the 5 mg/kg caffeine dose. Accordingly, if the study reported only the results from the first administration of caffeine, this participant would be considered as a non-responder. By contrast, if only the results from the second testing were reported, the same participant would be classified as a responder to caffeine. While working with limited data, these initial results imply a possible ‘learning effect’ for caffeine which needs to be considered when interpreting individual data.

## 5. Conclusions

As discussed herein, the estimate by Southward et al. [1] that there might be up to 33% of those that do not respond to caffeine ingestion might be an over-extrapolation of the current data. In fact, the number of those that do not respond to caffeine might be minimal given that using a different test of performance, changing the dose of caffeine, conducting repeated measures with the same test and the same dose of caffeine on different occasions (i.e., providing a ‘learning effect’ period), or possibly even adjusting the timing of caffeine ingestion based on genotype (as already nicely highlighted by Southward et al. [1]) might change a response to caffeine ingestion from a negative to a positive. To expand our current knowledge on the variation in responses to caffeine ingestion future studies should consider presenting the individual responses, and the interpretation of these responses should reflect some of the information presented herein.

## Figures and Tables

**Table 1:** Revised analysis of the prevalence of non-responders to the ergogenic effects of caffeine ingestion on exercise performance (based on the review by Southward et al. [1]).

Reference	Number of Non-Responders as Classified by Southward et al. [1]	Coefficient of Variation for the Performance Test	Number of Non-Responder While Factoring in the Range of the Error of the Measurement (for Studies That Provided These Data)
Acker-Hewitt et al. [3]	2/10	1.4%	0/10
Astorino et al. [4]	3/16	1.5%	1/16
Astorino et al. [5]	1/9	2.5%	0/9
Beaumont et al. [6]	1/8	Not reported	Unable to determine
Christensen et al. [7]	4/12	Not reported	Unable to determine
Church et al. [8]	8/20	Not reported	Unable to determine
Desbrow et al. [9]	3/9	Not reported	Unable to determine
Desbrow et al. [10]	4/16	Not reported	Unable to determine
Gonçalves et al. [11]	6/40	2.9%	4/40
Graham-Paulson et al. [12]	1/11	Not reported	Unable to determine
Guest et al. [13] 2 mg/kg	38/101	Not reported	Unable to determine
Guest et al. [13] 4 mg/kg	32/101	Not reported	Unable to determine
O’Rourke et al. [14]	3/30	Not reported	Unable to determine
Pitchford et al. [15]	2/9	Not reported	Unable to determine
Roelands et al. [16]	4/8	Not reported	Unable to determine
De Alcantara Santos et al. [17]	2/8	0.9%	1/8
Skinner et al. [18]	1/14	0.9%	0/14
Stadheim et al. [19]	2/10	3.2%	0/10
Stadheim et al. [20]	4/13	2%	0/13
Womack et al. [21]	3/35	Not reported	Unable to determine
Pooled number of participants and the number of non-responders			6/120 (5%)

## References

[1] Southward K., Rutherfurd-Markwick K., Badenhorst C., Ali A. (2018). The role of genetics in moderating the inter-individual differences in the ergogenicity of caffeine. Nutrients.

[2] Hopkins W.G. (2000). Measures of reliability in sports medicine and science. Sports Med..

[3] Acker-Hewitt T.L., Shafer B.M., Saunders M.J., Goh Q., Luden N.D. (2012). Independent and combined effects of carbohydrate and caffeine ingestion on aerobic cycling performance in the fed state. Appl. Physiol. Nutr. Metab..

[4] Astorino T.A., Cottrell T., Lozano A.T., Aburto-Pratt K., Duhon J. (2011). Ergogenic effects of caffeine on simulated time-trial performance are independent of fitness level. J. Caffeine Res..

[5] Astorino T.A., Roupoli L.R., Valdivieso B.R. (2012). Caffeine does not alter RPE or pain perception during intense exercise in active women. Appetite.

[6] Beaumont R.E., James L.J. (2016). Effect of a moderate caffeine dose on endurance cycle performance and thermoregulation during prolonged exercise in the heat. J. Sci. Med. Sport.

[7] Christensen P.M., Petersen M.H., Friis S.N., Bangsbo J. (2014). Caffeine, but not bicarbonate, improves 6 min maximal performance in elite rowers. Appl. Physiol. Nutr. Metab..

[8] Church D.D., Hoffman J.R., LaMonica M.B., Riffe J.J., Hoffman M.W., Baker K.M., Varanoske A.N., Wells A.J., Fukuda D.H., Stout J.R. (2015). The effect of an acute ingestion of Turkish coffee on reaction time and time trial performance. J. Int. Soc. Sports Nutr..

[9] Desbrow B., Barrett C.M., Minahan C.L., Grant G.D., Leveritt M.D. (2009). Caffeine, cycling performance and exogenous, CHO oxidation: A dose-response study. Med. Sci. Sports Exerc..

[10] Desbrow B., Biddulph C., Devlin B., Grant G.D., Anoopkumar-Dukie S., Leveritt M.D. (2012). The effects of different doses of caffeine on endurance cycling time trial performance. J. Sports Sci..

[11] Gonçalves L.S., Painelli V.S., Yamaguchi G., de Oliveira L.F., Saunders B., da Silva R.P., Maciel E., Artioli G.G., Roschel H., Gualano B. (2017). Dispelling the myth that habitual caffeine consumption influences the performance response to acute caffeine supplementation. J. Appl. Physiol..

[12] Graham-Paulson T.S., Perret C., Watson P., Goosey-Tolfrey V.L. (2016). Improvement of sprint performance in wheelchair sportsmen with caffeine supplementation. Int. J. Sports Physiol. Perform..

[13] Guest N., Corey P., Vescovi J., El-Sohemy A. (2018). Caffeine, CYP1A2 genotype, and endurance performance in athletes. Med. Sci. Sports Exerc..

[14] O’Rourke M.P., O’Brien B.J., Knez W., Paton C.D. (2008). Caffeine has a small effect on 5-km running performance of well-trained and recreational runners. J. Sci. Med. Sport.

[15] Pitchford N.W., Fell J.W., Leveritt M.D., Desbrow B., Shing C.M. (2014). Effect of caffeine on cycling time-trial performance in the heat. J. Sci. Med. Sport.

[16] Roelands B., Buyse L., Pauwels F., Delbeke F., Deventer K., Meeusen R. (2011). No effect of caffeine on exercise performance in high ambient temperature. Eur. J. Appl. Physiol..

[17] Santos Rde A., Kiss M.A., Silva-Cavalcante M.D., Correia-Oliveira C.R., Bertuzzi R., Bishop D.J., Lima-Silva A.E. (2013). Caffeine alters anaerobic distribution and pacing during a 4000-m cycling time trial. PLoS ONE.

[18] Skinner T.L., Jenkins D.G., Taaffe D.R., Leveritt M.D., Coombes J.S. (2013). Coinciding exercise with peak serum caffeine does not improve cycling performance. J. Sci. Med. Sport.

[19] Stadheim H.K., Kvamme B., Olsen R., Drevon C.A., Ivy J.L., Jensen J. (2013). Caffeine increases performance in cross-country double-poling time trial exercise. Med. Sci. Sports Exerc..

[20] Stadheim H.K., Nossum E.M., Olsen R., Spencer M., Jensen J. (2015). Caffeine improves performance in double poling during acute exposure to 2000-m altitude. J. Appl. Physiol..

[21] Womack C.J., Saunders M.J., Bechtel M.K., Bolton D.J., Martin M., Luden N.D., Dunham W., Hancock M. (2012). The influence of a CYP1A2 polymorphism on the ergogenic effects of caffeine. J. Int. Soc. Sports Nutr..

[22] Grgic J., Mikulic P. (2017). Caffeine ingestion acutely enhances muscular strength and power but not muscular endurance in resistance-trained men. Eur. J. Sport Sci..

[23] Churchward-Venne T.A., Tieland M., Verdijk L.B., Leenders M., Dirks M.L., de Groot L.C., van Loon L.J. (2015). There are no nonresponders to resistance-type exercise training in older men and women. J. Am. Med. Dir. Assoc..

[24] Jenkins N.T., Trilk J.L., Singhal A., O’Connor P.J., Cureton K.J. (2008). Ergogenic effects of low doses of caffeine on cycling performance. Int. J. Sport Nutr. Exerc. Metab..

[25] Astorino T.A., Cottrell T., Lozano A.T., Aburto-Pratt K., Duhon J. (2012). Increases in cycling performance in response to caffeine ingestion are repeatable. Nutr. Res..

